# Emergent Circularly Polarized Luminescence from Self-Assembled Chiral-Ligand-Functionalized CsPbBr_3_ Nanocrystals

**DOI:** 10.1021/acs.nanolett.6c02634

**Published:** 2026-09-11

**Authors:** Prasenjit Mandal, Mohit Chaudhary, Jeanne Cloarec, Oleg V. Prezhdo, Dmitry Baranov

**Affiliations:** † Division of Chemical Physics and NanoLund, Department of Chemistry, 5193 Lund University, P.O. Box 124, SE-221 00 Lund, Sweden; ‡ Department of Chemistry and Chemical Biology, 1104 University of New Mexico, Albuquerque, New Mexico 87106, United States; § ESPCI Paris, Université PSL, 10 rue Vauquelin, 75005 Paris, France

**Keywords:** Chirality transfer, circularly polarized luminescence, perovskite nanocrystals, nanocrystal superlattices, self-assembly, spin lifetime

## Abstract

Chiral surface ligands can impart optical activity to all-inorganic perovskite nanocrystals, but how this affects chiroptical properties of their assemblies remains unclear. Here we show that self-assembly of CsPbBr_3_ nanocubes synthesized with a small amount of added chiral ligand *N*-benzylcinchonidinium bromide (BCD-Br) results in measurable circularly polarized luminescence (CPL). BCD-Br-treated nanocrystals show weak circular dichroism but no detectable CPL in colloidal dispersion. Upon assembly into CsPbBr_3_ and mixed halide superlattices, they exhibit CPL with a luminescence dissymmetry factor, *g*_lum_, of (5 ± 2) × 10^–3^, whereas untreated controls remain near the noise level. Polarization-resolved transient absorption (TA) reveals an extended spin lifetime of hundreds of picoseconds in BCD-Br-treated nanocrystals, while linear-response time-dependent density functional theory (LR-TDDFT) calculations suggest that inter-nanocrystal coupling can enhance the chiroptical response. These results support the idea that ordered nanocrystal assembly can convert weak ligand-induced chirality into an emergent chiroptical response in perovskite nanocrystal solids.

## Graphical abstract

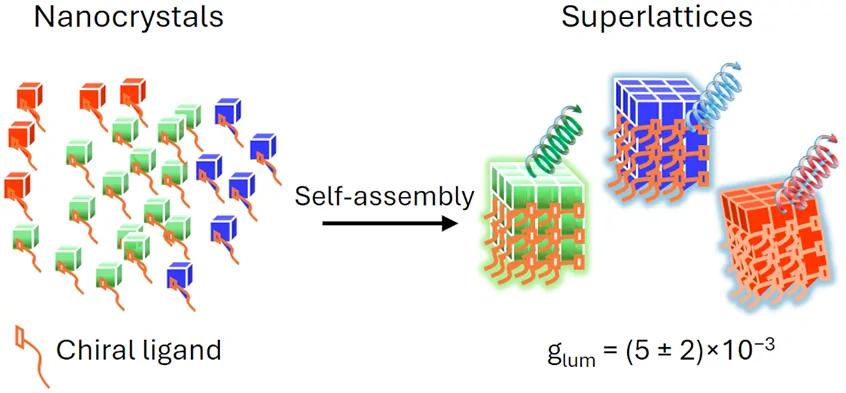

Chirality is a fundamental structural feature that governs optical activity, molecular recognition, and spin-selective processes in matter.^1,2^ An object is chiral if it does not coincide with its mirror image by any rotation or translation. In nanoscale materials, the emergence of chiroptical responses is particularly compelling because it enables the conversion of structural asymmetry into useful functions, such as circularly polarized light emission, circular dichroism, and spin-dependent interactions.^3–5^ CPL from semiconductor nanocrystals has therefore attracted considerable attention as a route toward advanced displays, optical encryption, enantioselective sensing, and spin-optoelectronic devices.^6,7^ Achieving strong, controllable CPL in colloidal nanocrystals remains challenging because the nanocrystal core is typically achiral, and the chiroptical response is often weak, transient, or highly sensitive to ligand and processing history.

Metal halide perovskite nanocrystals have emerged as an especially attractive platform for chirality transfer onto photophysics because of their high photoluminescence quantum yields, narrow emission line widths, tunable band gaps, and defect-tolerant electronic structure.^8–10^ These features make them promising candidates for CPL-active materials. In many cases, chirality is introduced at the surface through chiral ligands or by placing nanocrystals into external chiral matrices. The resulting optical activity often reflects local surface perturbations, such as structural distortions or matrix polarization properties.^11^ For example, Liu et al. showed the chiral absorption and emission properties of CsPbBr_3_ nanocrystals grafted on silica nanohelices.^12^ Kim et al. described CPL emission arising from chiral ligand binding to the nanocrystal surface.^13,14^ A single-particle investigation by Lv et al. reported that around 20% of individual chiral ligand-functionalized nanocrystals exhibit a measurable chiroptical response.^15^

Self-assembly offers a powerful route to bridge molecular-scale chirality and emergent nanoscale optical behaviors.^16^ When nanocrystals organize into superlattices, the interparticle spacing, orientational order, and collective electronic coupling can all be modulated in ways that are inaccessible in dispersed colloids. In this context, the self-assembly of nanocrystals bearing chiral ligands offers a potential route to enhanced hybridization between the ligands’ electronic states and those of the nanocrystals within the superlattice. Unlike molecular chiral fluorophores, whose optical activity originates from intrinsic structural asymmetry, chiral nanocrystal assemblies rely on collective organization to amplify chiroptical responses through interparticle interactions. Recent studies have demonstrated that ligand-induced self-assembly can serve as an efficient route for chirality transfer, leading to enhanced circularly polarized luminescence (CPL) in systems such as CdSe/ZnS quantum dots assembled with chiral gelators and CdTe nanoparticle assemblies.^17–20^ Similar strategies have started to be explored for perovskite nanocrystals.^12,21–23^

Ordered superlattices of perovskite nanocrystals attract considerable interest for their ability to exhibit emergent collective phenomena.^24–26^ These include superfluorescence, amplified spontaneous emission, long-range exciton diffusion, and collective diffraction.^27–30^ Several studies have demonstrated that ligand-mediated assembly can induce the formation of twisted microbelt structures from lead halide perovskite clusters and nanoplatelets, or promote oriented attachment into microscale nanowires, both of which exhibit CPL activity,^22,23,31–33^ including light amplification.^34^ Given the tunability of the surface chemistry of perovskite nanocrystals and their self-assembly into highly ordered superlattices,^35,36^ with evidence of electronic coupling,^37–41^ the question arises of whether self-assembly of nanocrystals functionalized with chiral molecules into such ordered superlattices can amplify chiroptical response compared with isolated particles. Here, we show that self-assembly of achiral perovskite nanocrystals synthesized with chiral ligands (further termed BCD-Br-treated nanocrystals) induces chiroptical response in the superlattice films, whereas nanocrystals in colloidal dispersion or self-assembled without chiral-ligand treatment show weak chiroptical properties and CPL near the noise level. These results suggest that self-assembly introduces chirality into BCD-Br-treated perovskite nanocrystal superlattices.

CsPbBr_3_ nanocrystals of uniform size for self-assembly experiments were synthesized following a modified hot-injection protocol adapted from recent literature,^42,43^ with a detailed synthetic description provided in the Experimental Section. Briefly, PbBr_2_ was dissolved in a mixture of oleic acid and oleylamine in 1-octadecene, forming a clear precursor solution at 175 °C. Subsequent rapid injection of cesium oleate-initiated nucleation and growth of the nanocrystals, followed by cooling to room temperature and purification to isolate the colloidal nanocrystals. For chiral ligand functionalization, ca. 0.10 molar equiv of BCD-Br (structure shown in Figure S1) was introduced together with the PbBr_2_ at the start of the synthesis. BCD-Br is a commercially available chiral bulky quaternary ammonium salt used as a chiral phase-transfer catalyst,^44,45^ with optical activity of [α]_D_^20^ −138 (*c* = 1, chloroform) as stated by the manufacturer. A measured circular dichroism (CD) spectrum shows a peak value of −54.5 mdeg at 333 nm (Figure S2). As-synthesized nanocrystals were isolated by centrifugation and dispersed in toluene for further characterization.

The structural and optical properties of both pristine and BCD-Br-treated nanocrystals are summarized in Figure 1. Transmission electron microscopy (TEM) images (Figure 1a,b) reveal the formation of monodisperse, cubic-shaped nanocrystals in both cases. The average particle sizes were determined to be 8.3 nm for the oleate/oleylammonium-capped nanocrystals and slightly smaller, 7.5 nm, for the BCD-Br-treated nanocrystals (insets in Figure 1a,b). The reduction in size upon introduction of the chiral ligand could be due to etching or BCD-Br influencing the nucleation and growth of nanocrystals, possibly through competitive surface binding and modification of precursor reactivity. Optical absorption and photoluminescence (PL) spectra (Figure 1c) show nearly identical features for both samples, with an absorption onset at ∼507 nm and a narrow emission peak centered at 512 nm. The presence of chiral ligands induces a small CD in the sample (Figure 1d), suggesting weak chirality transfer through surface modification.^13,14,46–48^ The presence of BCD-Br on the nanocrystal surface was confirmed by ^1^H NMR, with an estimated upper bound of 10% of the BCD-Br compared to the combined oleate/oleylammonium species in the nanocrystal dispersions prior to self-assembly (Figures S3 and S4).

**Figure 1:**
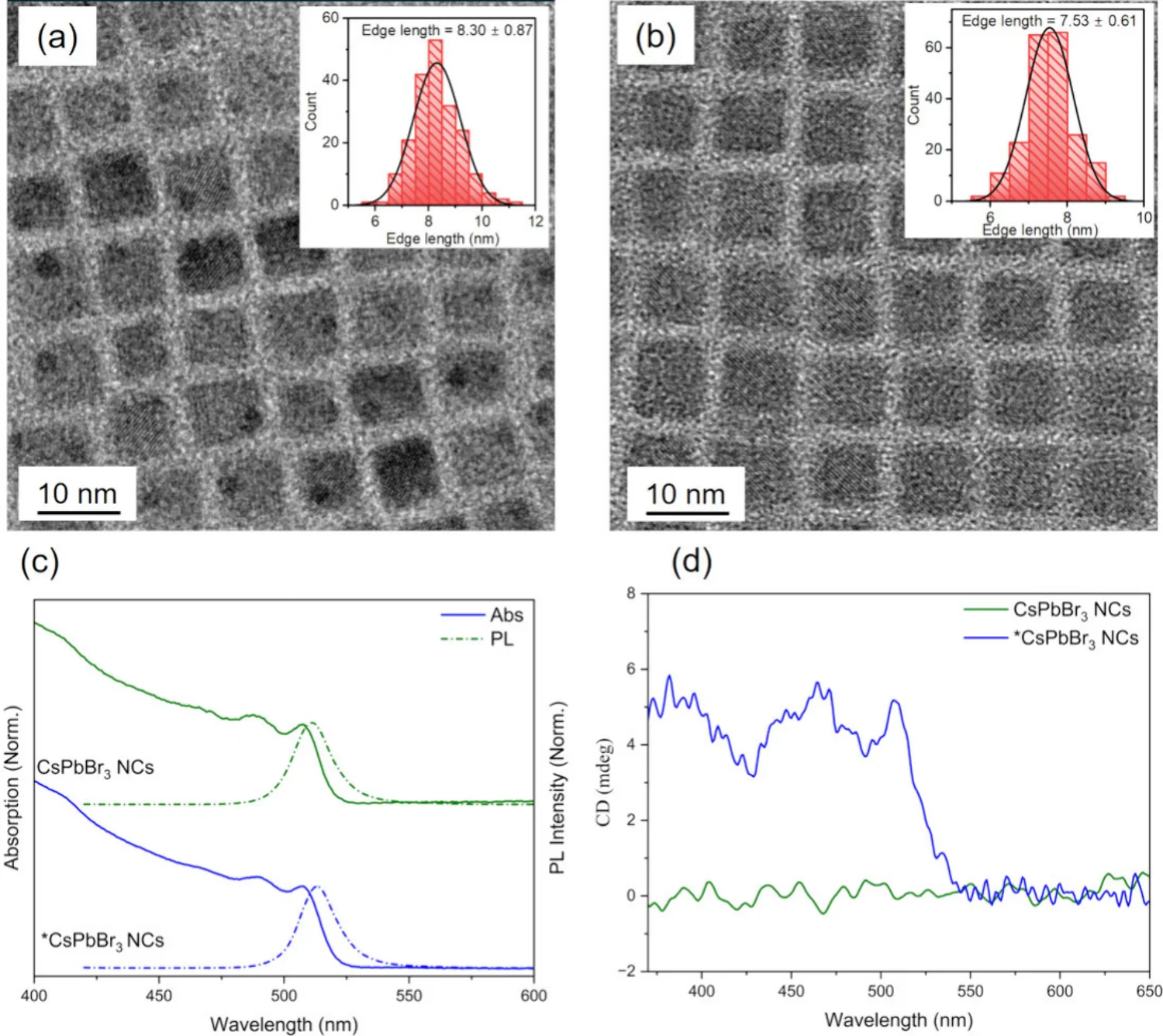
(a, b) Transmission electron microscopy images of (a) the oleate/oleylammonium-capped CsPbBr_3_ nanocrystals with average edge length 8.3 nm and (b) the BCD-Br/oleate/oleylammonium-capped nanocrystals with average edge length 7.5 nm. The insets show the histograms of the size distribution analysis. (c) Linear absorption (solid line) and PL (dashed line) spectra of nanocrystal samples. (d) CD spectra of nanocrystal samples. *CsPbBr_3_ indicates nanocrystals synthesized with BCD-Br.

## Superlattice Growth and Characterization

Next, we focus on the preparation of ordered superlattices from both achiral and chiral ligand-treated CsPbBr_3_ nanocrystals and systematically compare their structural and chiroptical properties. To achieve this, concentrated dispersions of CsPbBr_3_ nanocrystals in toluene were drop-cast onto glass substrates and allowed to dry slowly, typically inside a nitrogen-filled glovebox. Slow solvent evaporation leads to supersaturation, which increases nanocrystal concentration and promotes close packing of the nanocrystals in superlattices. The structural ordering of the resulting samples was probed with multilayer diffraction,^49,50^ using out-of-plane θ:2θ X-ray diffraction (XRD), as shown in Figure 2a. Superlattices grown from achiral and BCD-Br-treated nanocrystals display prominent (100) and (200) Bragg reflections at *q* ≈ 0.9–1.1 Å^–1^ and 2.0–2.2 Å^–1^, respectively, indicative of preferential orientation of the nanocrystals within the films. At lower scattering vectors (*q* < 0.8 Å^–1^), a periodic modulation of the diffraction intensity is observed, originating from the contrast in electron density between the inorganic nanocrystal cores and the surrounding organic ligand shell.^50^ Notably, the first-order Bragg peak exhibits clear splitting near ∼1 Å^–1^, which is characteristic of highly ordered multilayer structures with long-range coherence. These oscillations arise from constructive and destructive interference of X-rays scattered by periodically arranged nanocrystals and can be quantitatively described using a multilayer diffraction model. This approach, based on interference between neighbouring nanocrystal planes, enables the extraction of structural parameters such as crystal structure constant, interparticle spacing, and disorder, as established in previous studies.^49^

**Figure 2:**
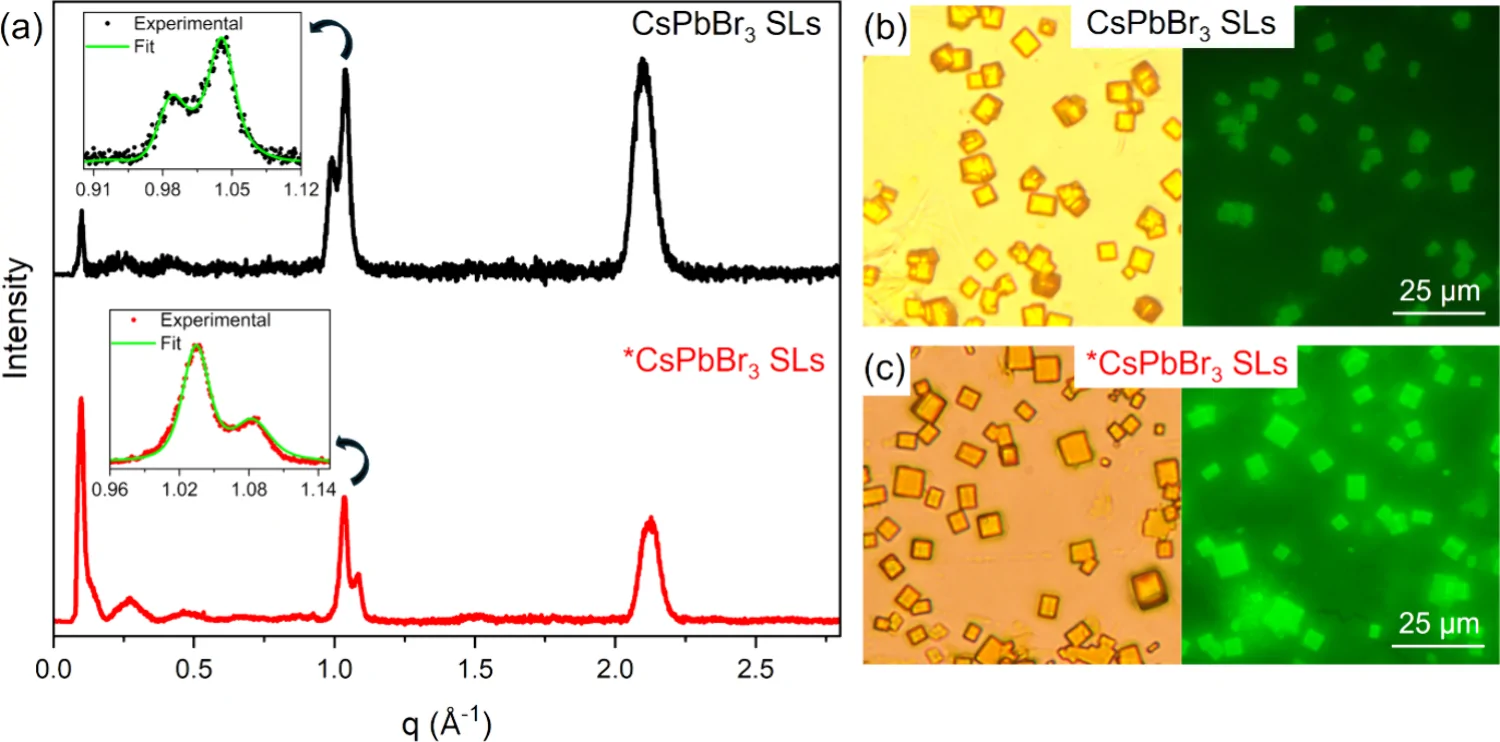
Characterization of nanocrystal superlattices film. (a) Out-of-plane θ:2θ XRD patterns of (bottom) BCD-Br/oleate/oleylammonium-capped CsPbBr_3_ nanocrystal superlattices and (top) standard oleate/oleylammonium-capped CsPbBr_3_ nanocrystal superlattices. The insets show the multilayer diffraction modulation of the first Bragg peak attesting to structural coherence of the superlattice samples. Structural parameters from the multilayer diffraction fit are discussed in the text. (b, c) Optical microscopy images of the superlattices grown on glass substrate under white light (left) and UV (right). See the text for definitions of structural parameters. SLs: superlattices.

Fitting the diffraction profiles (shown in the inset of Figure 2a) using this model yields key structural parameters, including the lattice constant (fixed at *d* = 5.837 Å), superlattice periodicity (Λ), interparticle spacing (*L*), stacking disorder (σ_*L*_), nanocrystal size (*N*, expressed as the number of atomic planes), and size distribution (σ_*N*_). For the superlattices made from achiral CsPbBr_3_ nanocrystals, the extracted values are Λ = 109.10 Å and *L* = 39.05 Å with a relatively narrow size distribution (*N* = 13, σ_*N*_ = 1.55) and moderate stacking disorder (σ_*L*_ = 1.49 Å), consistent with prior characterization of similar samples.^36,49^ The superlattices made from BCD-Br-treated nanocrystals exhibit reduced periodicity (Λ = 94.97 Å), smaller interparticle spacing (*L* = 36.60 Å), reduced average size and larger size distribution (*N* = 11, σ_*N*_ = 4.84), and reduced stacking disorder (σ_*L*_ = 1.20 Å). The reduced periodicity is consistent with the smaller size of BCD-Br-treated nanocrystals from TEM (Figure 1). The reduced interparticle spacing can be rationalized by partial incorporation of BCD-Br into the interparticle ligand environment (Note S1). Optical microscopy images of the superlattice films (Figure 2b,c) reveal the formation of well-defined rectangularly shaped superlattices with lateral dimensions ranging from 3 to 10 μm in both cases. Both samples exhibit bright PL under UV illumination in the optical microscope (Figure 2b,c). Visual comparison of microscopy images did not reveal substantial differences in superlattice morphologies between the samples.

## Spin Relaxation Dynamics of Nanocrystals and Superlattice Films

To study the effect of a chiral ligand on optical properties, exciton spin dynamics in achiral and chiral CsPbBr_3_ nanocrystals and superlattices were compared using ultrafast TA spectroscopy. TA measurements were performed by tracking the differential absorption as a function of pump–probe delay time. Near-resonant left- and right-circularly polarized (σ^–^ and σ^*+*^) ca. 100 fs pump pulses at 400 nm, with a fluence of 8 μJ/cm^2^, were used to selectively generate spin-polarized excitons. The spin-dependent relaxation of the photoexcited carrier population was monitored with a broadband circularly polarized probe in either the co-circular or cross-circular configuration, as illustrated in Figure 3a for the case of the σ^–^ pump. The representative evolution of the transient signals for co-circular (σ^–^σ^–^) and cross-circular (σ^–^σ^*+*^) configurations for chiral samples is presented in Figure 3b (that for achiral samples is shown in Figures S5 and S6), and data for the σ^*+*^ pump are provided in Figures S7 and S8. Achiral and chiral samples exhibit comparable spectral profiles under these excitation conditions, characterized by a pronounced negative ground-state bleach (GSB) centered at the excitonic absorption peak (ca. 507–511 nm), along with two positive photoinduced absorption features located on the higher-energy and lower-energy sides of the bleach. The overall spectral response near the band edge can be attributed to a combination of transient Stark effects, arising from photoinduced local electric fields and band filling.^51,52^

**Figure 3:**
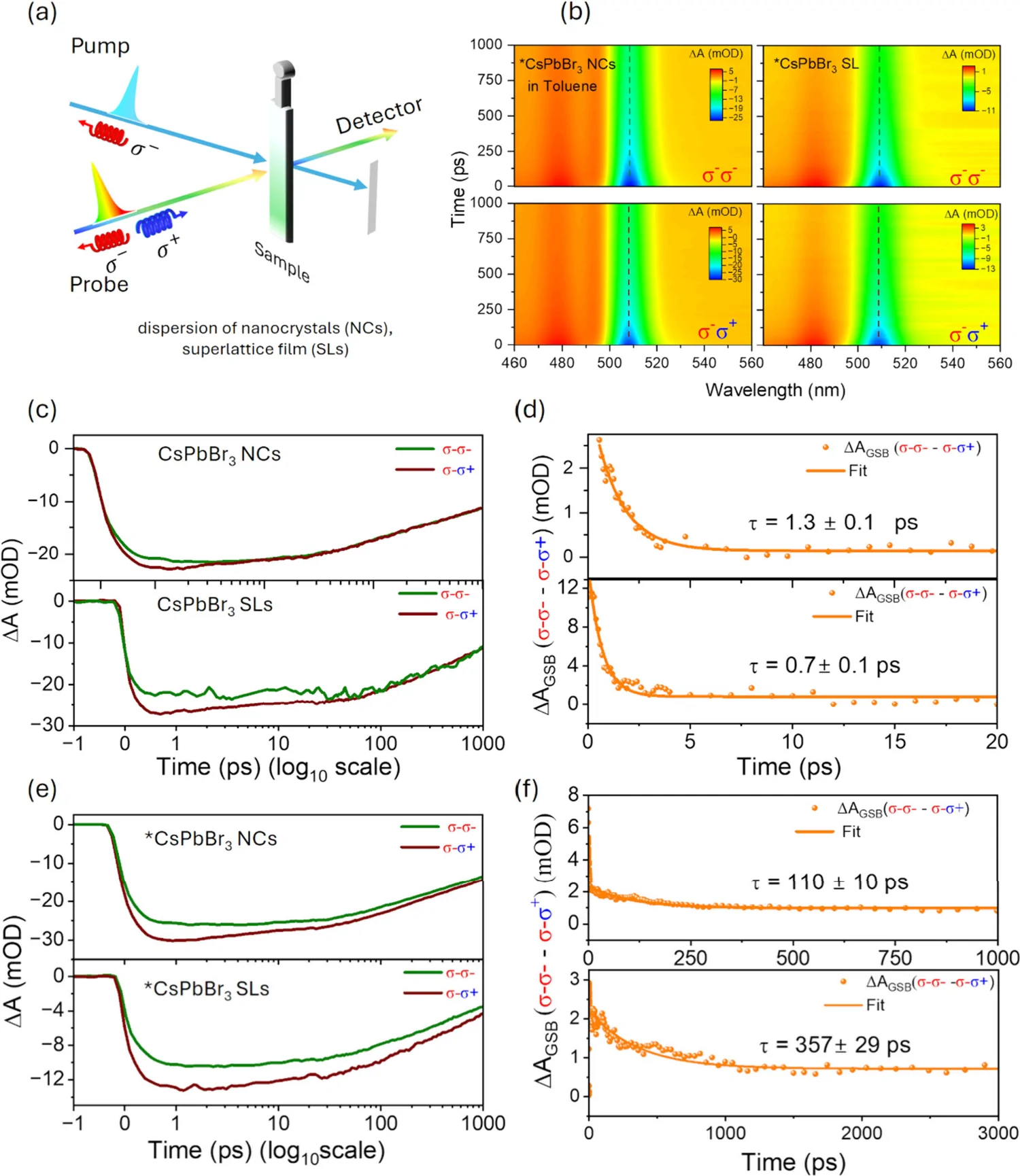
Polarization-resolved TA of spin dynamics in achiral and BCD-Br-treated CsPbBr_3_ nanocrystal dispersions and superlattice films. (a) Schematic of the TA experiment: σ^–^ and σ^*+*^ denote left- and right-circular polarization, respectively. Samples were excited at 400 nm with a fluence of 8 μJ/cm^2^ and probed with broadband white light. (b) TA maps of BCD-Br-treated CsPbBr_3_ nanocrystals in toluene and the corresponding superlattice film, measured for co-circular (σ^–^σ^–^) and cross-circular (σ^–^σ^*+*^) pump–probe configurations. Dashed vertical lines indicate the probe wavelengths used to extract the dynamics. (c) Polarization-dependent GSB dynamics of achiral CsPbBr_3_ nanocrystals in toluene and in the corresponding superlattice film. (d) Spin-polarization dynamics obtained from the difference between co- and cross-circular TA signals in (c), fitted with a single-exponential decay plus a constant offset to extract the spin-relaxation lifetime, τ. (e, f) Analogously, GSB and spin dynamics for BCD-Br-treated nanocrystals in toluene and in the superlattice film.

To quantify the influence of chirality on spin relaxation, we analyzed the temporal evolution of the GSB under both polarization configurations (Figure 3c,e) following previously reported procedures for spin dynamics analysis.^53–55^ Briefly, the spin relaxation lifetime was extracted by first calculating the polarization-dependent difference signal, [Δ*A*_co-circular_(*t*) – Δ*A*_cross‑circular_(*t*)], from two TA datasets recorded with the same circularly polarized pump and opposite probe helicities. The resulting decay was then fitted with a single-exponential function plus a constant offset. The co-circular (σ^–^σ^–^) dynamics includes contributions from both spin relaxation and population decay, whereas the cross-circular (σ^–^σ^+^) response primarily reflects spin-independent relaxation pathways. By subtracting the latter from the former, we isolate the exciton spin relaxation dynamics, as shown in Figure 3d,f. In both systems, the extracted spin dynamics exhibits an initial rapid rise on the picosecond time scale, followed by a decay. Fitting the decay component with a single-exponential function yields the characteristic spin lifetime.^53–55^

For achiral CsPbBr_3_ nanocrystals in dispersion and superlattice film, the extracted spin lifetime is between 1 and 3 ps (Figure 3d and Table S1), indicating rapid spin depolarization. This behavior originates from the strong spin-orbit coupling associated with heavy Pb atoms, which facilitates efficient spin mixing and accelerates spin relaxation via electron-hole exchange interactions and scattering processes.^56,57^ As a result, the initially generated spin polarization decays quickly, leading to a minimal distinction between co- and cross-circular signals at longer delay times. In contrast, BCD-Br-treated nanocrystals showed a prolonged spin lifetime of 100s ps (Figure 3f and Table S1). We hypothesize that increased spin lifetime in the BCD-Br-treated nanocrystals is a result of the chirality transfer from the ligand to nanocrystals, which we further discuss below.

## Circularly Polarized Luminescence

The CPL of both individual nanocrystals and their assembled superlattices is shown in Figure 4. The CPL was quantified using the photoluminescence dissymmetry factor, defined as $g_{\mathrm{lum}}=2\frac{I_{R}-I_{L}}{I_{R}+I_{L}}$ _,_ where *I*_R_ and *I*_L_ are the emission intensities of the right- and left-circularly polarised light, respectively. The PL intensities of LCP and RCP are shown in Figure S9. As shown in Figure 4a,b, neither pristine CsPbBr_3_ nanocrystals nor those treated with chiral ligands exhibit any measurable CPL signal, indicating nondetectable intrinsic chiroptical activity in colloidal dispersions. In contrast, a distinct CPL response emerges upon assembly. The superlattices formed from chiral-ligand-functionalized nanocrystals show an average *g*_lum_ of (5 ± 2) × 10^–3^ (Figure 4d and Table S2), whereas the CPL signal from superlattices films of achiral CsPbBr_3_ nanocrystals is near the detection limit, ca. 1 × 10^–3^ (Figure 4c and Note S2).^58^ Regarding the absolute magnitude of the obtained *g*_lum_ value, it falls in the low-medium range. It is higher than those for some ligand-treated systems (e.g., 6 × 10^–4^ for (*R*/*S*)-3MPEA-functionalized CsPbBr_3_ ^59^ and 1 × 10^–3^ for l/d-tryptophan-functionalized CsPbBr_3_ ^60^). It is lower compared to those of nanocubes in chiral matrices (e.g., 8.39 × 10^–3^ in chiral MOFs^61^) and much lower that those of nanocrystal films integrated with metasurfaces (e.g., (1.6–3) × 10^–1^)^62^ or assembled with liquid crystal templates (e.g., (0.3–2.4) × 10^–1^).^63^ This leaves clear scope to further develop the approach through choice of ligand, control of the assembly, and its combination with other materials.

**Figure 4:**
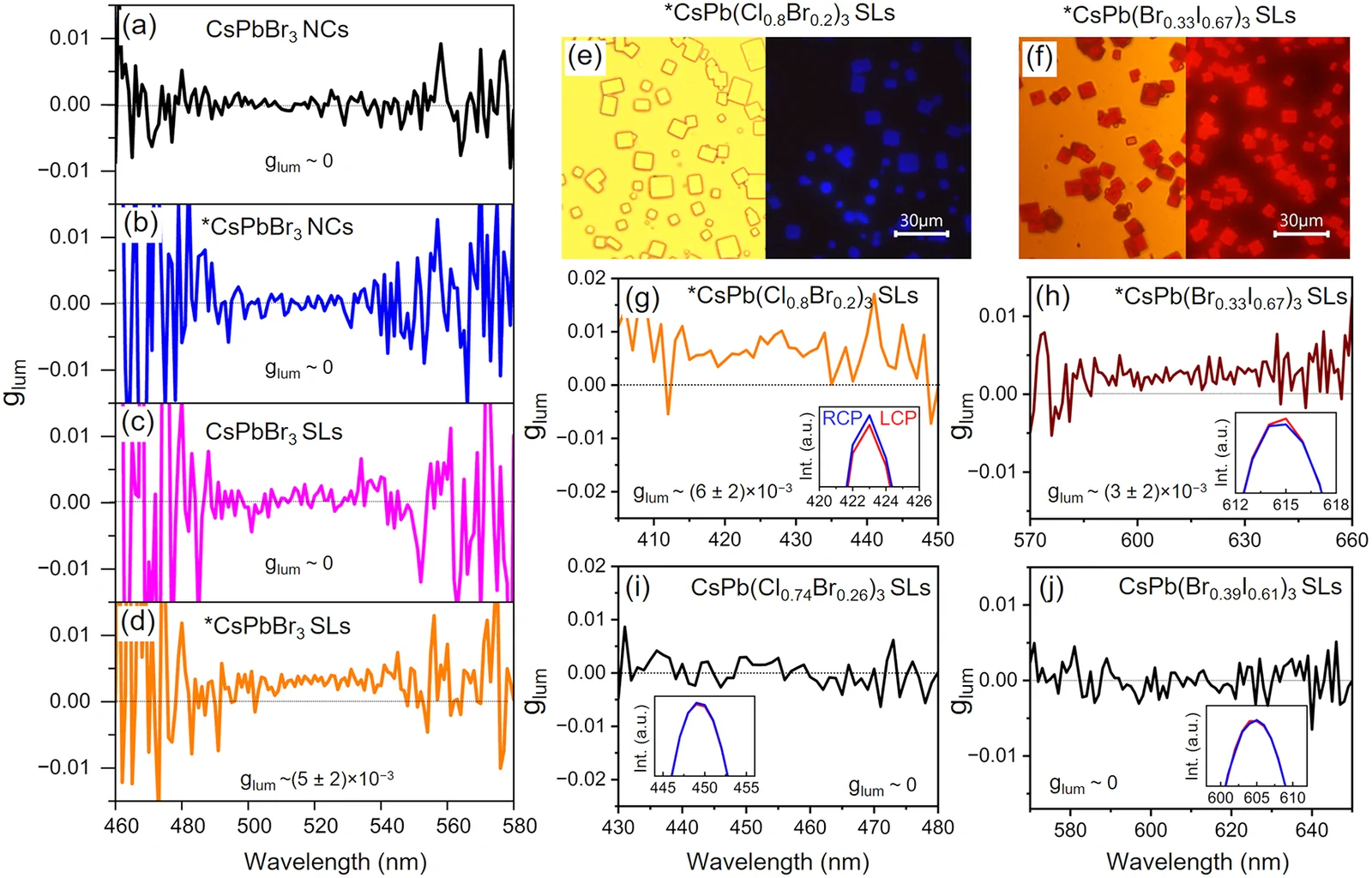
CPL characterization of chiral and achiral (control) perovskite nanocrystals and their superlattice films. (a, b) CPL spectra of chiral and achiral CsPbBr_3_ nanocrystals dispersed in toluene. (c, d) CPL spectra of the corresponding CsPbBr_3_ superlattice films on glass. (e, f) White-light (left) and UV (right) optical microscopy images of chiral CsPb­(Cl_0.8_Br_0.2_)_3_ and CsPb­(Cl_0.8_Br_0.2_)_3_ superlattice films. (g–j) CPL spectra of mixed halide chiral superlattice films and their achiral controls. CPL measurements were performed using 405 nm excitation for CsPbBr_3_ and CsPb­(Br_0.33_I_0.67_)_3_ and 370 nm excitation for CsPb­(Cl_0.8_Br_0.2_)_3_.

To test the applicability of the BCD-Br approach to other halide compositions, we studied analogous superlattices prepared by anion exchange of CsPbBr_3_ nanocrystals with solid PbCl_2_ and PbI_2_ to obtain CsPb­(Cl_0.8_Br_0.2_)_3_ and CsPb­(Br_0.33_I_0.67_)_3_ compositions, followed by self-assembly (Figure 4e,f). Both mixed halide systems exhibit CPL signals with *g*_lum_ values of (6 ± 2) × 10^–3^ and (3 ± 2) × 10^–3^, respectively (Figure 4g,h). In contrast, control superlattices prepared from the nanocrystals without BCD-Br show signal at the noise level (Figure 4i,j). These results demonstrate that the CPL response is consistently correlated with the introduction of BCD-Br into the synthesis of nanocrystals across different halide compositions and self-assembly.

To support the experimental observations of chirality transfer and gain mechanistic insight, we calculated the absorption and CD spectra of model CsPbBr_3_ nanocrystal monomers and dimers, with and without a chiral molecule, using LR-TDDFT (Figure 5). The nanocrystal was modeled as a 2 × 2 × 2 CsPbBr_3_ cube comprising eight unit cells. For the chiral nanocrystal, one surface Cs^+^ ion was replaced by an (*R*)-methylbenzylammonium (*R*-MBA) molecule (Figure 5a), which was used as a computationally tractable proxy for BCD^+^. A similar atomistic approach using chiral molecules bound to the surface of CsPbBr_3_ nanocrystals has previously been employed to investigate chirality transfer and the resulting chiroptical response.^64^ A chiral nanocrystal dimer, used here as a minimal proto-superlattice model, was constructed by stacking two chiral nanocrystal monomers. The use of nanocrystal dimers as minimal models for probing inter-nanocrystal electronic coupling and delocalization in perovskite nanocrystal assemblies is motivated by previous theoretical studies.^65^

**Figure 5:**
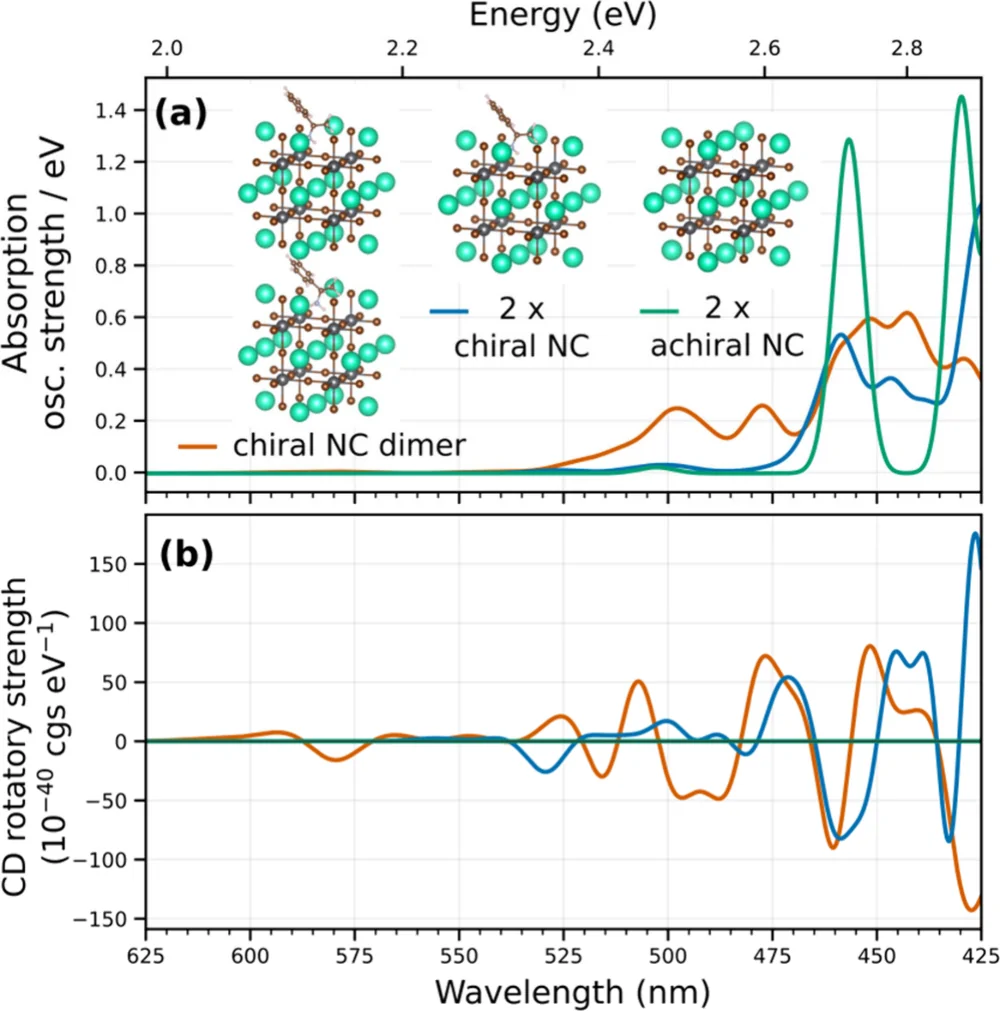
(a) Absorption and (b) circular dichroism spectra of the achiral nanocrystal, chiral nanocrystal monomer, and chiral nanocrystal dimer. The spectra of the achiral and chiral nanocrystal monomers are multiplied by a factor of 2 to enable direct comparison with the dimer.

The monomer, dimer, and achiral nanocrystals retain comparable HOMO-aligned electronic gaps, with only a modest reduction in the dimer (see Figure S10), indicating that dimerization does not strongly alter the band-edge electronic structure. This is consistent with the experimentally observed absence of substantial spectral shifts between the superlattice films and nanocrystal dispersions. Figure 5a,b compares the calculated spectra of the chiral dimer with twice the spectra of the isolated chiral and achiral monomers. This provides a qualitative microscopic comparison between an interacting chiral nanocrystal assembly and the corresponding independent nanocrystals. Notably, the dimer exhibits a nonadditive chiroptical response, with inter-nanocrystal coupling redistributing both oscillator and rotatory strengths relative to two independent monomers. This redistribution leads to enhanced CD for several low-energy excitations. Because these states are expected to contribute to emission following excited-state relaxation, they may also be relevant to the experimentally observed CPL. Although the dimer provides only a minimal representation of the superlattice, the calculations suggest that coupling between closely packed chiral nanocrystals can enhance the chiroptical response beyond that of independent nanocrystals, providing a plausible microscopic origin for the experimental observations.

To summarize, while nanocrystals in dispersion, even when functionalized with chiral ligands, do not exhibit detectable CPL, their assembled counterparts display a clear CPL response, underscoring the importance of the collective mesoscale organization. Overall, this work reports the observation of self-assembly-mediated chirality transfer, which can serve as a route to generating chiroptical functionality from initially achiral nanocrystal building blocks. Given the diversity of colloidal nanocrystals, functionalization with chiral molecules followed by controlled assembly into superstructures offers a broadly applicable strategy for chirality transfer. This approach potentially provides a versatile framework for designing materials with tunable circularly polarized emission for future applications in spin optoelectronics, chiral photonics, and quantum light technologies.

## Supplementary Material



## Data Availability

The data underlying this study are openly available in Zenodo at 10.5281/zenodo.21877178.

## Notes

Experimental and computational details, molecular structure, and solution CD spectrum of BCD-Br; ^1^H NMR characterization of chiral and achiral CsPbBr_3_ nanocrystals; TA spectra of as-synthesized and BCD-Br-treated CsPbBr_3_ nanocrystals and superlattice films; CPL spectra of nanocrystal dispersions and superlattice films; additional control experiments; calculated total density of states for achiral, chiral, and dimer nanocrystals (PDF)
