# Supplementary material for: Emergent Circularly Polarized Luminescence from Self-Assembled Chiral-Ligand-Functionalized CsPbBr3 Nanocrystals
Source: Nano Lett. 2026 Sep 11;26(38):12716–24. doi: 10.1021/acs.nanolett.6c02634 (PMC13632589; doi:10.1021/acs.nanolett.6c02634)
Supplement: Supplementary file 1 [file nl6c02634_si_001.pdf]

Supporting information for

Emergent Circularly Polarized Luminescence from Self-Assembled Chiral-Ligand-Functionalized $CsPbBr_3$ Nanocrystals

Prasenjit Mandal[1*], Mohit Chaudhary[2], Jeanne Cloarec[3], Oleg V. Prezhdo[2], Dmitry Baranov[1*]

[1]Division of Chemical Physics and NanoLund, Department of Chemistry, Lund University, P.O. Box 124, Lund, SE-221 00 Sweden

[2]Department of Chemistry and Chemical Biology, University of New Mexico, Albuquerque, New Mexico 87106, United States

[3]ESPCI Paris, Université PSL, 10 rue Vauquelin, 75005 Paris, France

*Corresponding authors: prasenjit.mandal@chem.lu.se , dmitry.baranov@chem.lu.se

Contents:

Experimental Details

Computational Details

Figure S1. Structure of the BCD-Br molecule

Figure S2. CD and absorption spectra of BCD-Br in chloroform

Figure S3. ^{1}H-NMR spectra of BCD-Br

Figure S4. Comparison of ^{1}H-NMR spectra of achiral, chiral NCs and pure BCD-Br

Note S1 on XRD *L* contraction

Figures S5, S6. Transient absorption spectra and GSB dynamics of as-synthesized (achiral) $CsPbBr_3$ nanocrystals and SL films

Figures S7, S8. Transient absorption spectra and GSB dynamics of as-synthesized BCD-Br treated $CsPbBr_3$ nanocrystals and SL films

Figure S9. CPL spectra of nanocrystal dispersions and superlattice films of $CsPbBr_3$

Note S2 on control CPL experiments

Figure S10. Comparison of TDOS for various nanocrystals by LD-TDDFT

Tables:

S1. Spin lifetime of nanocrystal dispersions and superlattice films of $CsPbBr_3$.

S2. g_{lum} for $CsPb(Cl/Br)_3$, $CsPbBr_3$ and $CsPb(Br/I)_3$ superlattice films.

Experimental Details

Chemicals

Cs_2CO_3 (99%, Sigma-Aldrich), $PbBr_2$ (≥98%, Sigma-Aldrich), PbI_2 (99.999% trace metals basis, Sigma-Aldrich), $PbCl_2$ (powder 98%, Sigma-Aldrich) *N*-benzylcinchonidinium bromide (BCD-Br, 97%, Sigma-Aldrich), 1-octadecene (ODE, 90%, Sigma-Aldrich), oleic acid (OA, 90%, Sigma-Aldrich), oleylamine (OLAM, 70%, Sigma-Aldrich), toluene (99.8%, anhydrous, Sigma-Aldrich), and chloroform-d (99.8 atom % D, Sigma-Aldrich) were used as received without further purification.

Synthesis of Cs-oleate

Cs-oleate was prepared by dissolving Cs_2CO_3 (200 mg) in 875 µL of OA and 7.5 mL of ODE at 120 °C under a nitrogen atmosphere for 1 h.

Synthesis of $CsPbBr_3$ and BCD-Br-Treated $CsPbBr_3$ Nanocrystals

Achiral $CsPbBr_3$ nanocrystals were prepared following a literature method with slight modifications. All syntheses were carried out in 20 mL glass vials positioned in an aluminum heating block placed on a hot plate, with the block preheated to 185 °C. A precursor mixture consisting of $PbBr_2$ (72 mg), 1-octadecene (ODE, 5 mL), oleic acid (OA, 50 µL), and oleylamine (OLAM, 500 µL) was heated to 175 °C until the $PbBr_2$ was completely dissolved. The vial was then removed from the heating block and allowed to cool under constant stirring. When the temperature reached 163-167 °C, 500 µL of Cs-oleate solution was rapidly injected, leading to an immediate color change to greenish-yellow and the formation of $CsPbBr_3$ nanocrystals. The reaction mixture was subsequently cooled to room temperature under continuous stirring in ambient conditions.

The crude product was purified by centrifugation at 4000 rpm for 3 min, followed by removal of the supernatant. The precipitate was subjected to an additional centrifugation step (4000 rpm, 1 min), after which residual solvent was carefully decanted. A final centrifugation (4000 rpm, 3 min) was performed to ensure complete removal of remaining liquid. The purified nanocrystals were redispersed in 1 mL of toluene, centrifuged once more, and the resulting supernatant was passed through a 0.2 µm PTFE syringe filter prior to further use.

For the preparation of BCD-Br-treated $CsPbBr_3$ nanocrystals, 9 mg of BCD-Br was introduced in the synthesis together with the Pb precursor ($PbBr_2$), while all other synthetic and purification steps remained unchanged.

Synthesis of $CsPb(Br_xI_{1-x})_3$ and BCD-Br-Treated $CsPb(Br_xI_{1-x})_3$ Nanocrystals via Anion Exchange

$CsPbBr_3$ nanocrystals were first synthesized following the procedure described above. After Cs-oleate injection, the reaction mixture was cooled to room temperature. Subsequently, PbI_2 (0.4 mmol, ca. 184 mg) was added directly to the crude reaction mixture, which was then stirred at room temperature for 2 h to facilitate anion exchange.

The resulting nanocrystals were collected by centrifugation at 4000 rpm for 3 min, and the supernatant was discarded. The precipitate was subjected to an additional centrifugation step at 4000 rpm for 2 min, after which the residual solvent was carefully removed. The purified nanocrystals were then redispersed in 2 mL of toluene and centrifuged again at 4000 rpm for 3 min to remove unreacted precursors. The resulting supernatant was passed through a 0.2 μm PTFE syringe filter. Finally, the filtered nanocrystal dispersion was concentrated to approximately 1 mL under a gentle N_2 flow and used for subsequent experiments.

9 mg of BCD-Br was introduced together with the $PbBr_2$ precursor, while all other synthetic steps remained unchanged in the case of chiral ligand functionalized $CsPb(Br_xI_{1-x})_3$.

Synthesis of $CsPb(Cl_xBr_{1-x})_3$ and BCD-Br-Treated $CsPb(Cl_xBr_{1-x})_3$ Nanocrystals via Anion Exchange

Pristine and BCD-Br-treated $CsPbBr_3$ nanocrystals were first synthesized according to the procedures described above. Following Cs-oleate injection, the reaction mixture was cooled to room temperature and centrifuged at 4000 rpm for 3 min. The supernatant was discarded, and the remaining fraction was subjected to an additional centrifugation step at 4000 rpm for 2 min. The resulting precipitate was redispersed in 5 mL of toluene.

Then $PbCl_2$ (120 mg), oleic acid (OA, 20 μL), and oleylamine (OLAM, 20 μL) were added to the nanocrystal dispersion (same conditions for regular and BCD-Br-treated batches). The mixture was stirred at room temperature for 6 h to promote anion exchange. After completion of the reaction, the dispersion was centrifuged to remove insoluble material, and the resulting supernatant was filtered through a 0.22 μm PTFE syringe filter. Finally, the purified nanocrystal dispersion was concentrated to approximately 1 mL under a gentle N_2 flow and used for subsequent experiments.

Self-assembly of Nanocrystals into Superlattices

Superlattice films of $CsPbBr_3$ and mixed-halide nanocrystals, both pristine and chiral ligand-functionalized, were prepared by drop-casting 30 μL of a concentrated toluene dispersion onto glass (pre-cut pieces of 1 mm-thick microscope slides or 0.15 mm-thick microscope cover slips) or silicon substrates placed inside a Petri dish within a nitrogen-filled glovebox. The solvent was allowed to evaporate slowly overnight, after which the resulting films were further characterized.

Characterization

Absorption and photoluminescence (PL) measurements were performed on dilute nanocrystal dispersions in toluene, prepared by diluting 20 µL of the stock solution in 3 mL of solvent. All optical measurements were conducted in a 10 mm path-length UV-quartz cuvette. All CPL measurements were carried out using a commercial OLIS DSM245 CPL spectrometer (OLIS Inc., USA). Depending on the spectral region of interest, excitation was provided by either a 370 nm or a 405 nm LED source. The diameter of the LED beam at the sample is ≈1.5 cm, which covers the entire SL film (1 cm x 1 cm). The PL signal was collected in a 90° geometry perpendicular to the excitation beam to minimize scattered excitation light. For measurements on the films, the sample was placed at ca. 35° incidence angle with respect to the surface normal. The emitted light was analyzed using a photoelastic modulator (PEM) in combination with a linear polarizer before being dispersed by a grating monochromator and detected with a photon-counting module. Data acquisition, instrument control, and spectral analysis were performed using OLIS SpectralWorks software. The instrument determines CPL by measuring the left- and right-circularly polarized emission components through a differential photon-counting approach synchronized with the PEM modulation. This method enables direct calculation of the luminescence dissymmetry factor (g_{lum}) from the measured I_{RCP} and I_{LCP} signals while minimizing systematic errors.

The wavelength axis of the monochromator was calibrated using a Hg-Ar calibration lamp by the manufacturer, and the wavelength-dependent response of the detection system was corrected using the manufacturer-provided emission standards and calibration procedures. For all measurements reported in the main text and supporting information, the samples were excited through the glass substrate (beam path: air-glass-sample-detector), while additional control experiments were performed by exciting from the sample side. For $CsPbBr_3$ samples, CPL spectra were recorded over the wavelength range of 450–580 nm using an integration time of 10 s per point. For mixed halides, the spectral range was adjusted accordingly to capture their PL spectra. The detection monochromator slit width was adjusted to provide a spectral resolution of approximately 0.12 nm, and spectra were collected at a scanning rate of 5 nm per minute. According to the manufacturer, the OLIS system should be able to resolve g_{lum} values down to the fifth decimal place (g_{lum} of 10^{-5}). That might be the case for photostable molecular systems in solution. In our experience with solid-state nanocrystal samples and polycrystalline materials, g_{lum} values on the order of 10^{-3} can be distinguished from achiral counterparts with a typical noise floor on the order of 10^{-4}.

For transmission electron microscopy (TEM), 10 µL of a concentrated nanocrystal dispersion was drop-cast onto a Lacey carbon-coated copper grid (300 mesh) and allowed to dry under ambient conditions. Bright-field TEM images were collected using a JEOL 3000F microscope operated at 300 kV. X-ray diffraction (XRD) patterns of the superlattice films were recorded in reflection geometry using a STOE STADI MP diffractometer with Cu Kα radiation (λ = 1.54 Å). Optical microscopy characterization of the superlattices was performed using a Kern OBN 148 microscope equipped with a digital camera (ODC 861) and UV illumination.

Transient absorption (TA) spectroscopy was conducted using a home-built femtosecond pump-probe setup. Laser pulses (796 nm, 100 fs, 4 kHz, 8 W) from a Solstice amplifier (Spectra Physics), seeded by a Mai Tai SP oscillator, were split to pump two optical parametric amplifiers (TOPAS-C, Light Conversion). One generated 400 nm pump pulses, while the other produced 1350 nm pulses that were focused onto a CaF_2 crystal to generate a broadband supercontinuum probe. The relative polarization of pump and probe beams was set to orthogonal linear states using a Berek compensator. A mechanical delay stage provided temporal control of the pump-probe delay up to 10 ns. Achromatic quarter-wave plates (Thorlabs, AQWP05M-600) were used to generate left- and right-circularly polarized pump and probe lights. The probe beam was divided into signal and reference paths, with the signal overlapping the pump at the sample position. Both beams were dispersed using a prism spectrograph and detected with a dual photodiode array (512 elements each, Pascher Instruments).

^{1}H NMR spectra were acquired on a 400 MHz spectrometer. NMR samples were prepared by dissolving the nanocrystal precipitate obtained after the final purification step in 0.6 mL of $CDCl_3$. In the case of the BCD-Br molecule, 15 mg of BCD-Br was dissolved in 0.6 mL of $CDCl_3$.

Computational details

Methods. The bulk $CsPbBr_3$ structure was first optimized using density-functional theory (DFT) as implemented in VASP, with the Perdew-Burke-Ernzerhof (PBE) generalized-gradient approximation for the exchange-correlation functional.[1, 2] The nanocrystal structure was subsequently carved from the relaxed bulk geometry. To construct the chiral nanocrystal model, one surface Cs ion was replaced by an *R*-methylbenzylammonium (R-MBA) molecule. The R-MBA molecule was then allowed to relax while the Cs, Pb, and Br atoms of the perovskite framework were kept fixed. The chiral nanocrystal dimer was constructed by stacking two chiral nanocrystal monomers, followed by an additional structural relaxation in which the perovskite atoms were again held fixed. The resulting optimized structures were used for the subsequent optical calculations.

Absorption and electronic circular dichroism (ECD) spectra were calculated using the ORCA 6.1.1 quantum-chemistry package.[3] Ground-state DFT calculations employed the PBE exchange-correlation functional[2] together with the minimally augmented ma-def2-SVP basis set.[4] Excited-state properties were calculated using full linear-response time-dependent density functional theory (LR-TDDFT) within the Casida formalism,[5] without invoking the Tamm-Dancoff approximation. To reduce the computational cost, the Casida excitation space was restricted to occupied molecular orbitals lying within 9 eV below the HOMO and virtual orbitals extending up to 6 eV above the HOMO. Absorption and ECD spectra were obtained from the calculated excitation energies and corresponding transition properties.

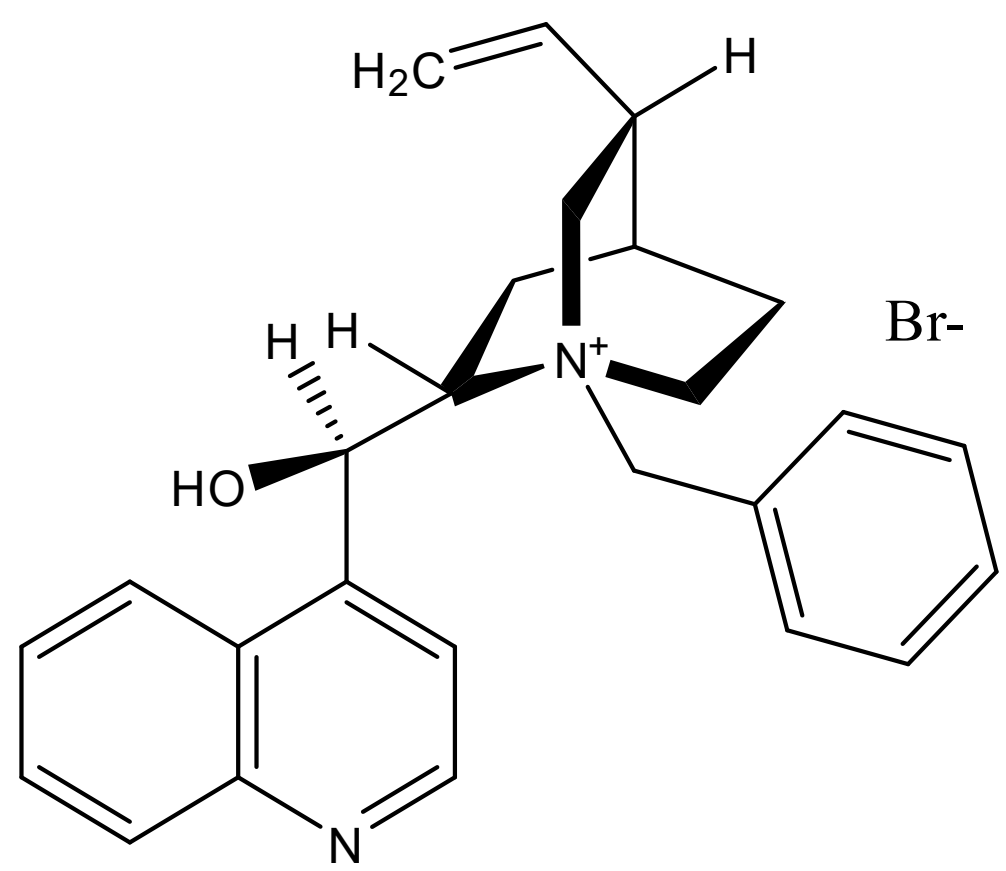

Figure S1. Structure of the chiral organic ligand of N-benzylcinchonidinium bromide (BCD-Br).

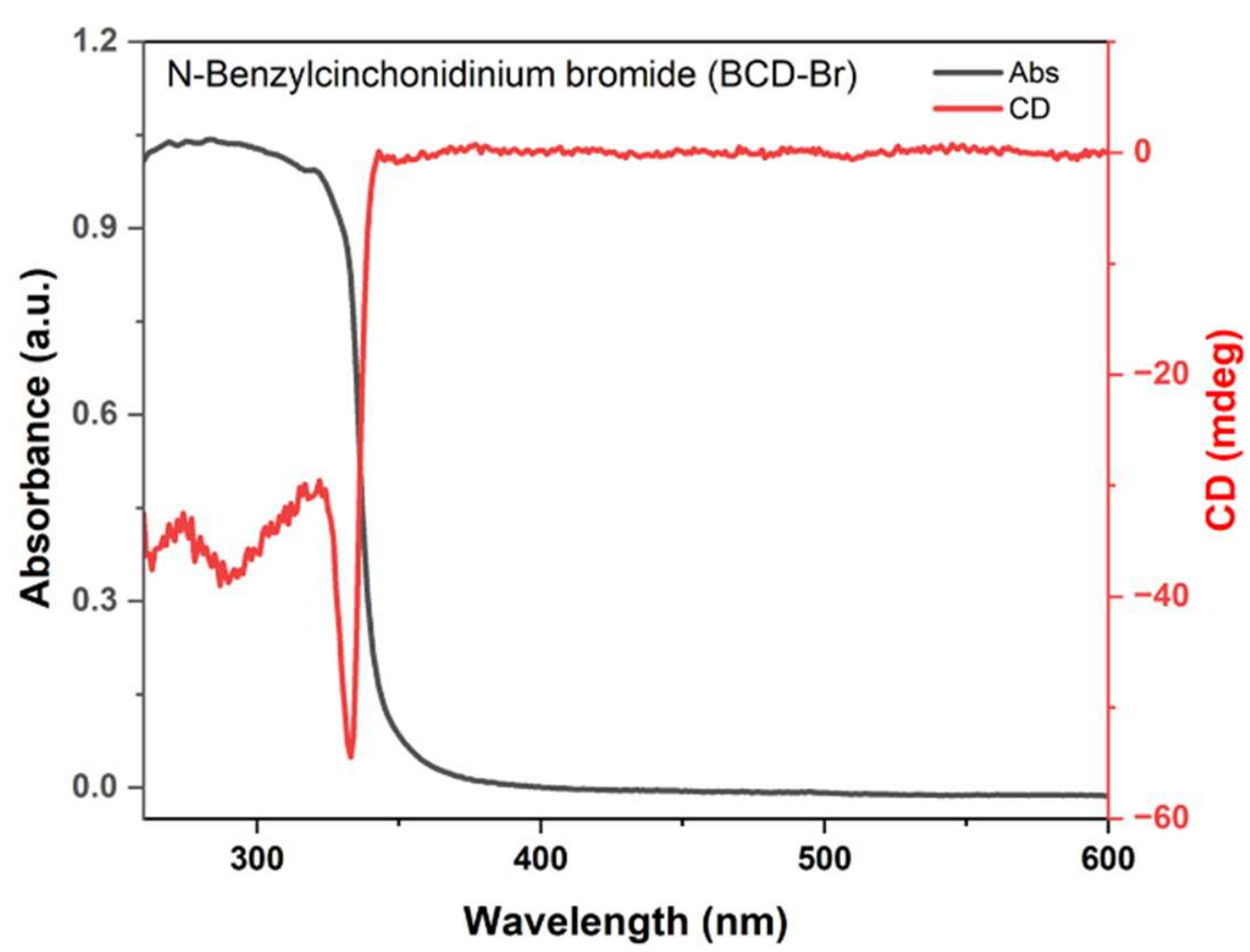

Figure S2. Absorption and circular dichroism (CD) spectra of 25 mg/mL BCD-Br solution in chloroform ($CHCl_3$).

BCD-Br Binding to the Nanocrystal Surface

In **Figures S3** and **S4** we compare the assigned spectrum of BCD-Br in $CDCl_3$ with that of the ^{1}H-NMR spectra of reference nanocrystals (i.e., without BCD-Br) and the nanocrystals synthesized in the presence of BCD-Br. The nanocrystal sample was prepared by drying the toluene dispersion used for assembly and redispersing the residue in $CDCl_3$.

The BCD-Br resonances in the BCD-Br-treated nanocrystal sample show both upfield and downfield shifts relative to free BCD-Br, indicating that the molecular environment of BCD-Br is perturbed in the presence of $CsPbBr_3$ nanocrystals. The upfield shifts of protons close to the quaternary ammonium center, especially those assigned to positions #11, #15, and #16, and to a lesser extent #20, suggest a change in the local ion-pairing or electrostatic environment around the quaternary nitrogen. This may be consistent with partial replacement or perturbation of the Br^- counterion environment by nanocrystal surface species, oleate, or bromide-rich surface sites. The resonances in the aromatic region remain comparatively sharper than the lower-field aliphatic/cage resonances. This suggests that the ammonium-containing part of BCD-Br has stronger interaction or reduced mobility in the nanocrystal sample compared to the aromatic groups. This differs from previously reported didodecyldimethylammonium-treated $CsPbBr_3$ nanocrystals[6], where surface-bound molecules produced broad resonances.

The approximate 1:18 ratio of the integral of the narrow multiplet at 8.80 ppm from BCD-Br (one proton bound to carbon #1) and the integral of the broad peak at ca. 5.33 ppm (two vinyl hydrogens from the double bonds in oleate and oleylammonium) can be interpreted as 1 BCD-Br molecule to 9 combined oleate/oleylammonium species in the nanocrystal dispersion used for self-assembly. Because the superlattices are prepared by solvent evaporation, all the low vapor pressure organics (ODE, BCD-Br, oleate/oleylammonium species) remain in the sample, either incorporated inside the superlattices or as the organic residue outside. This sets an upper bound of 10% BCD-Br in the superlattice film relative to the combined BCD-Br plus oleate/oleylammonium species.

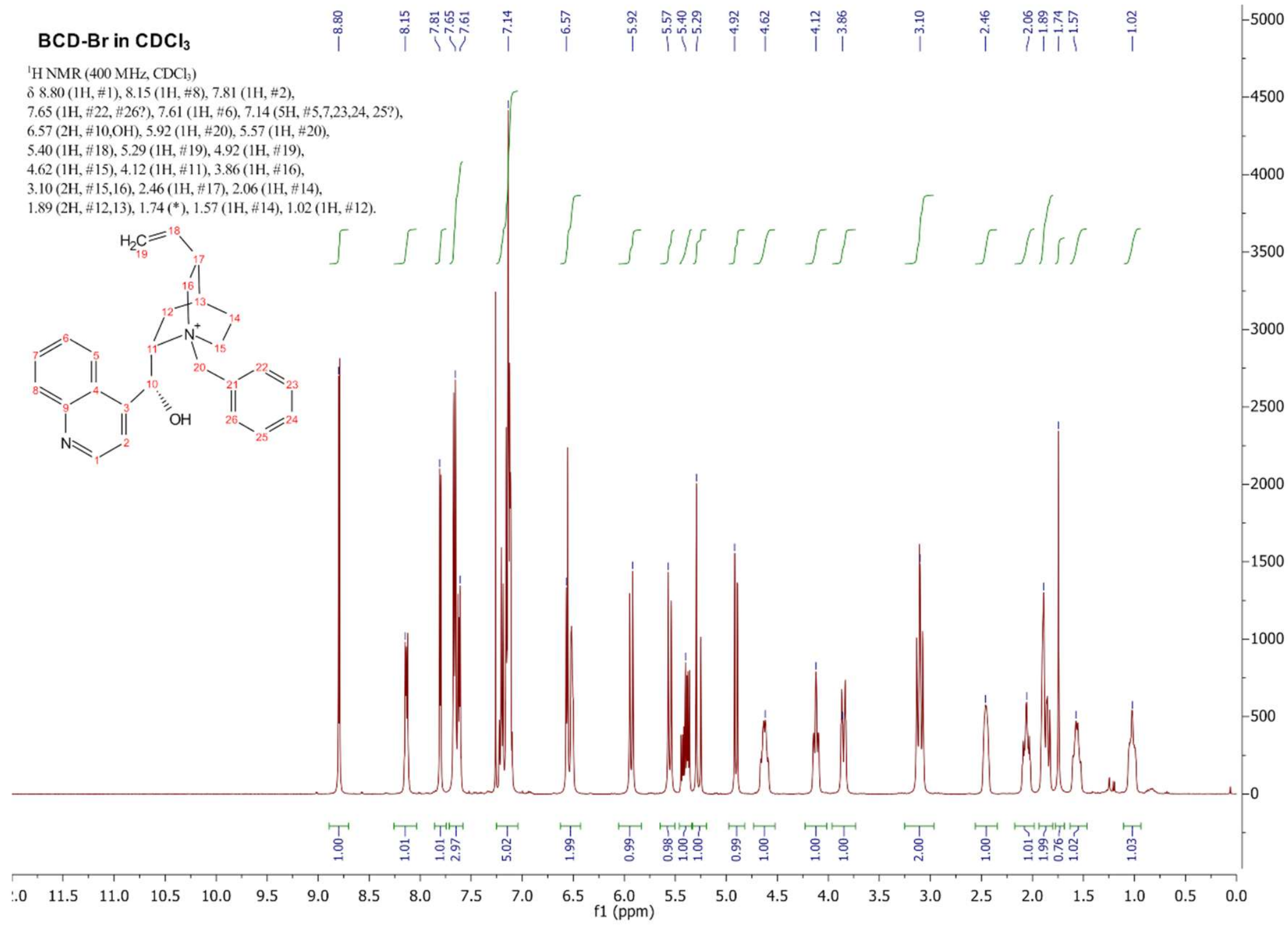

Figure S3. ^{1}H-NMR spectrum of BCD-Br in $CDCl_3$. The assignment of resonances follows earlier work.[7]

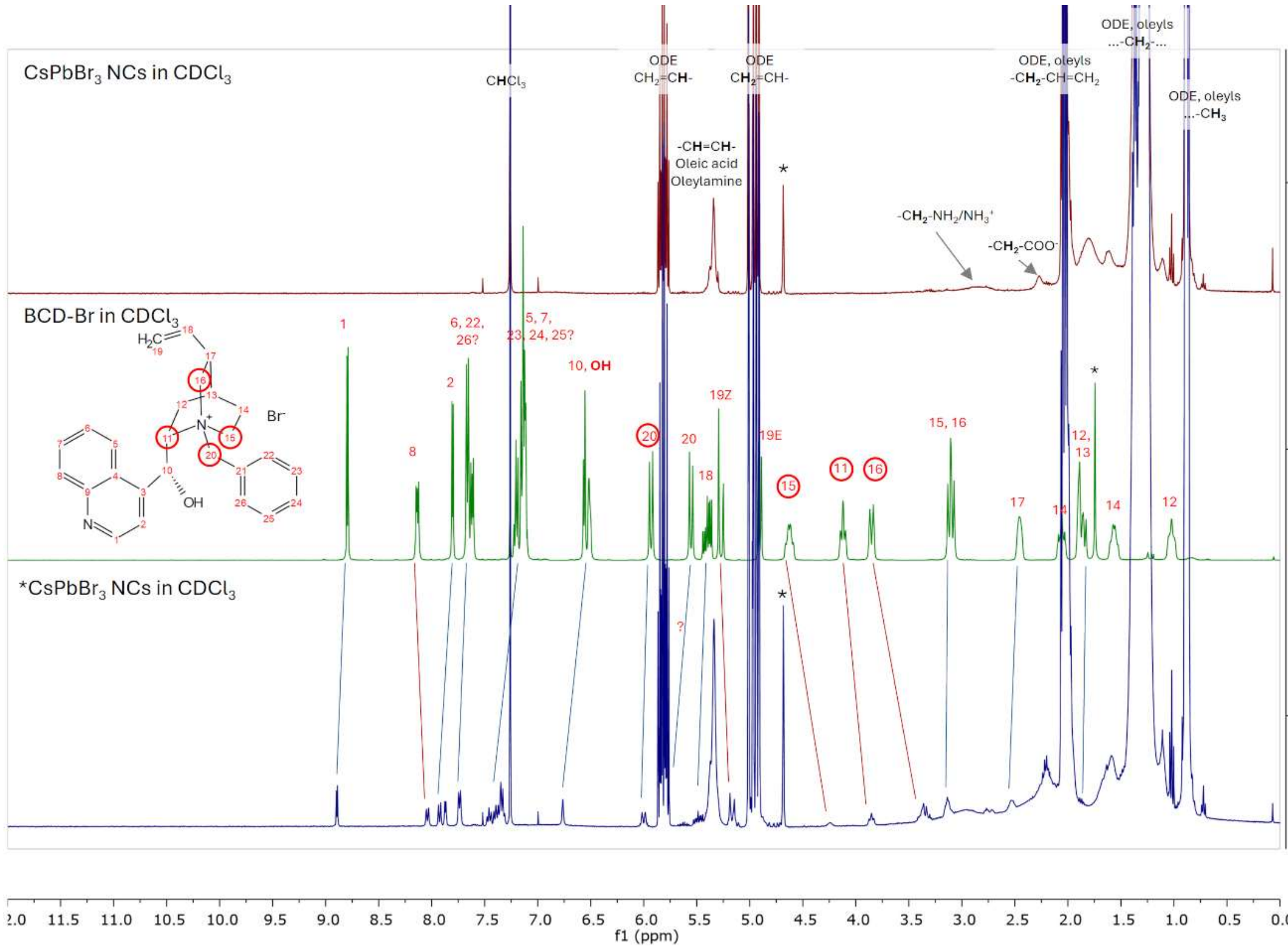

Figure S4. Comparison of ^{1}H-NMR spectra recorded in $CDCl_3$ for $CsPbBr_3$ nanocrystals synthesized without BCD-Br (top), free BCD-Br (middle), and BCD-Br-treated $CsPbBr_3$ nanocrystals (*$CsPbBr_3$, bottom; *$CsPbBr_3$ denotes nanocrystals synthesized in the presence of BCD-Br). The BCD-Br resonances are assigned according to the numbering shown in the molecular structure. Chemical-shift changes between free BCD-Br and the BCD-Br-treated nanocrystal sample are marked by blue lines for downfield shifts and red lines for upfield shifts. The asterisk marks a sharp singlet at 4.68 ppm observed also in the control nanocrystal sample and assigned to an unidentified impurity.

Note S1 on XRD *L* contraction

The out-of-plane XRD on superlattice films and corresponding multilayer diffraction analysis are sensitive to nanocrystal order and quantitative information about the contents of interparticle space, e.g., swelling with solvent molecules of tetrachloroethylene.[8]

The comparison of XRD patterns and fitting shown in **Figure 2** in the main text indicates two changes: smaller nanocrystal size for nanocrystals synthesized with BCD-Br (N= 11 vs 13, i.e. smaller on average by 2 lattice planes, ca. 1.2 nm edge length), and interparticle spacing L, shorter by ca. 2.4 Å (from 39 Å to 36.6 Å, approximately 6% contraction) in the superlattices made of nanocrystals synthesized with BCD-Br. From ^{1}H NMR, we estimated an upper bound of 10% of BCD-Br in the ligand environment of nanocrystals. Naively, if 100% of oleate/oleylammonium bilayer results in 39 Å separation, then interparticle separation in the BCD-Br-treated superlattices can be represented as 36.6 Å = 0.9 × 39 Å + 0.1 × **X** Å. Solving for **X = 15** Å. This is compatible with the dimensions of the cation of BCD-Br, based on its single-crystal structure.[9] Hence, the possibility that BCD-Br goes in between nanocrystals, partially replacing oleate/oleylammonium, is consistent with the shrinking of the interparticle distance.

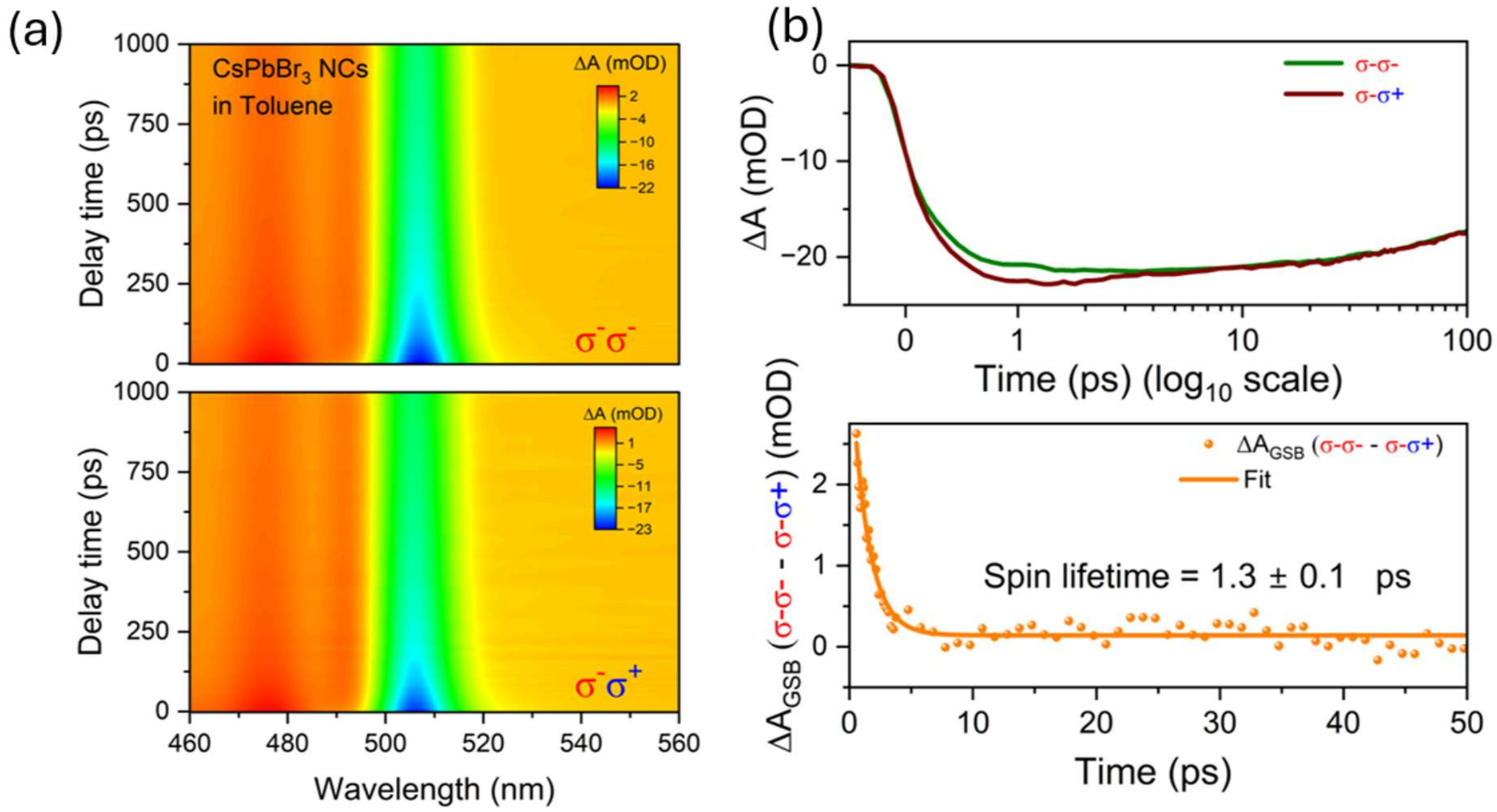

Figure S5. (a) Transient absorption spectra and (b)GSB dynamics of achiral $CsPbBr_3$ nanocrystal in toluene with LCP pump.

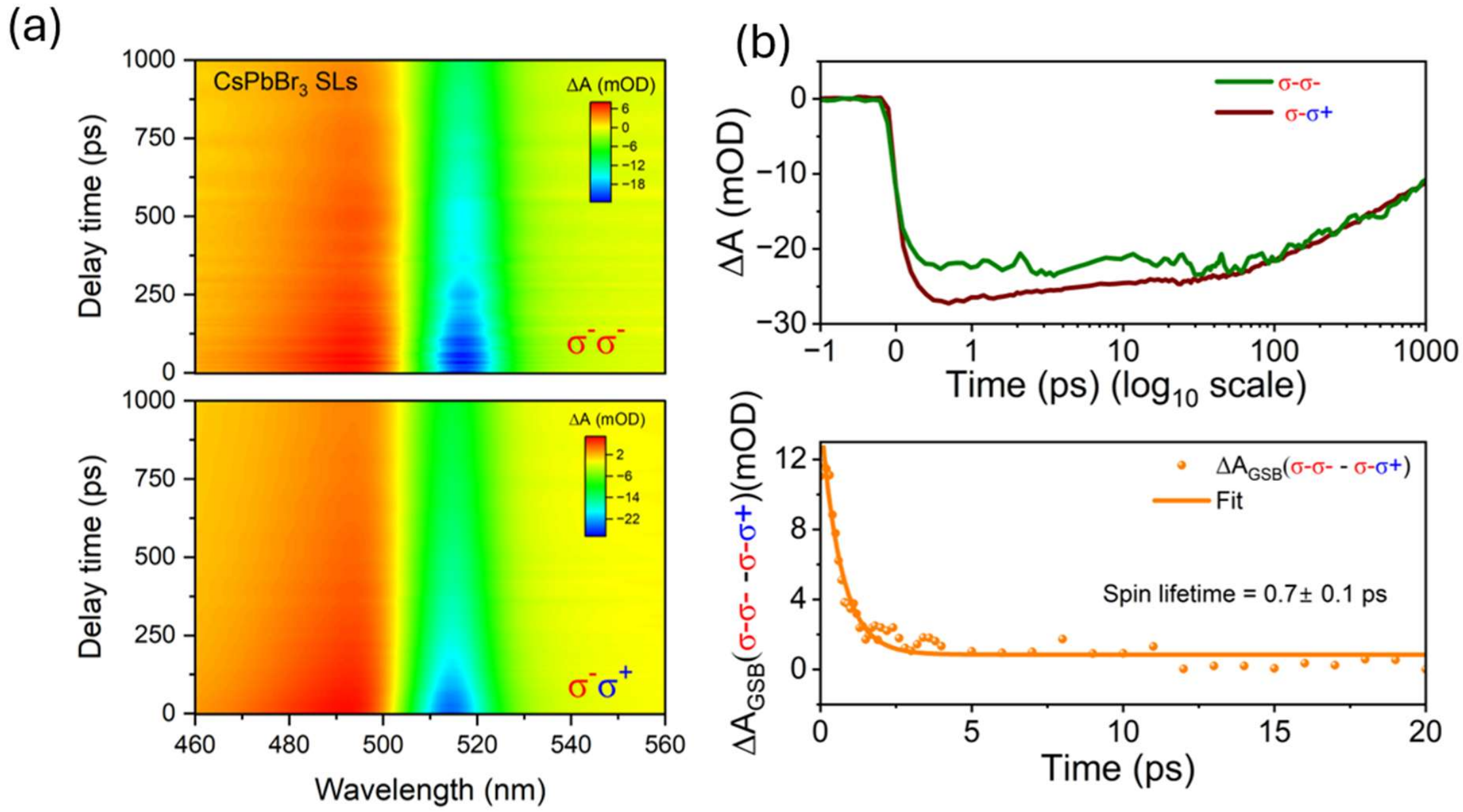

Figure S6. (a) Transient absorption spectra and (b) GSB dynamics of achiral $CsPbBr_3$ nanocrystal superlattices with LCP pump.

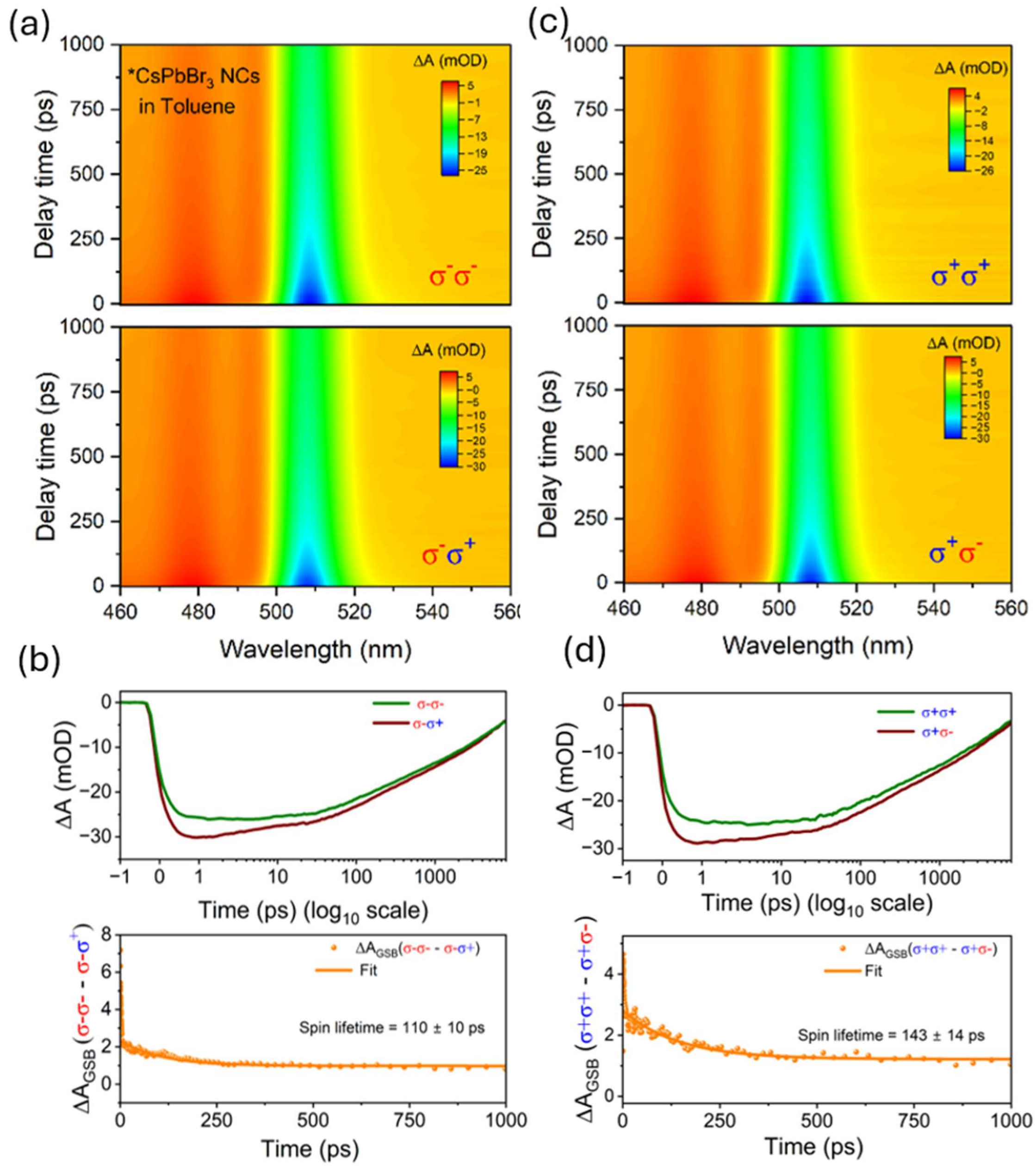

Figure S7. Transient absorption spectra and GSB dynamics of *$CsPbBr_3$ nanocrystals in toluene with (a,b) LCP pump and (c,d) RCP pump.

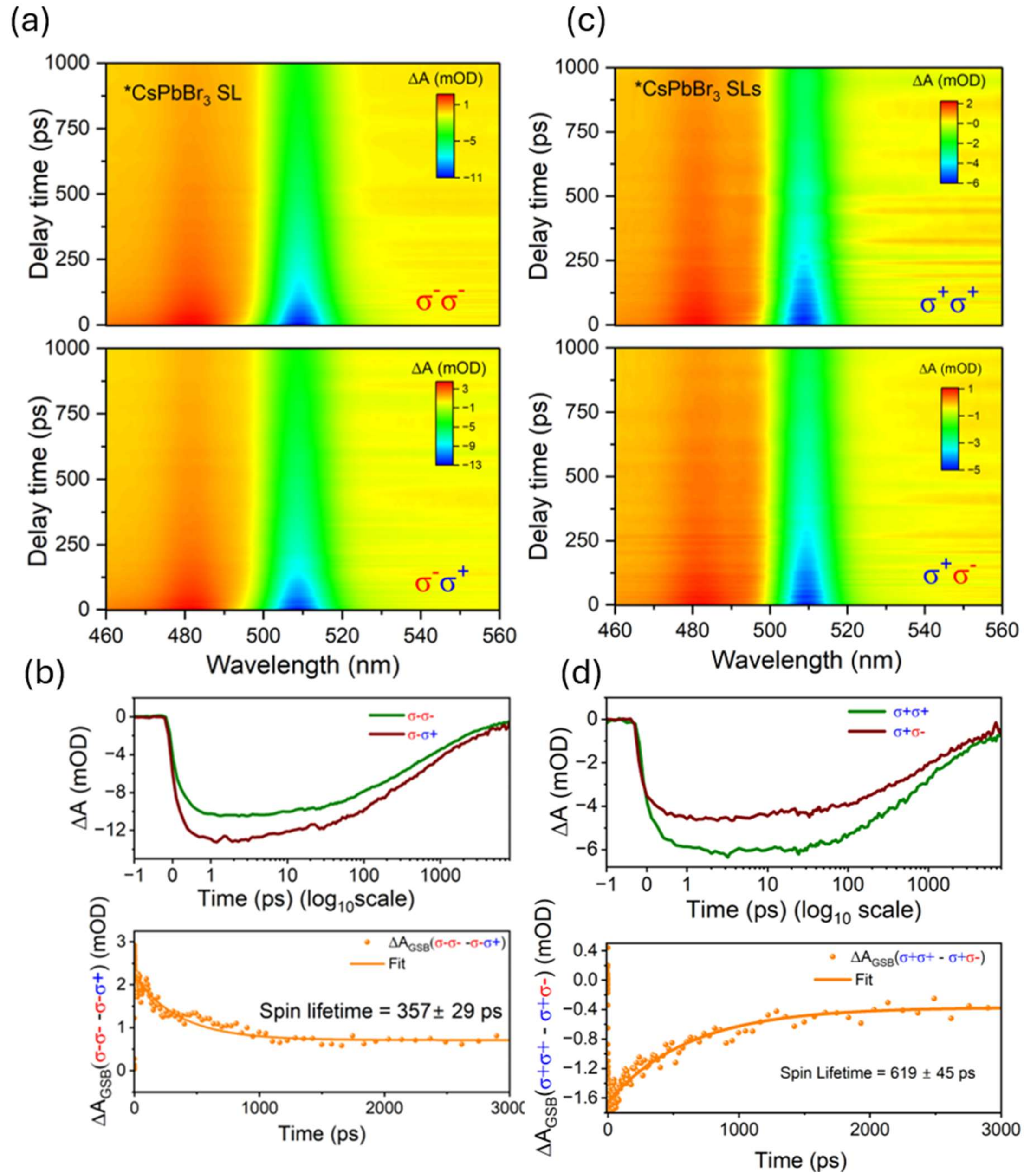

Figure S8. Transient absorption spectra and GSB dynamics of *$CsPbBr_3$ nanocrystal superlattice films with (a,b) LCP pump and (c,d) RCP pump.

Table S1. Spin relaxation lifetimes obtained by difference between ground state bleach (GSB) dynamics between co-/counter-circularly polarized pump-probe beam configurations. RCP – right-circularly polarized, LCP – left-circularly polarized. The spin relaxation lifetime was fit with a single-exponential decay. In all experiments on films, the pump and probe beams were incident on the sample side. Pump fluence 8 μJ/cm^2. Only pump polarization is mentioned, as for each pump polarization, TA was measured with both LCP and RCP probe. Superlattice films were immersed in inert silicone oil to minimize pump/probe scattering by reducing refractive index mismatch between the superlattice film and the surrounding medium.

**Sample**	**GSB peak wavelength**	**Pump polarization**	**Spin lifetime**
*$CsPbBr_3$ NCs in toluene	507 nm	LCP Pump	110 ± 10ps
*$CsPbBr_3$ NCs in toluene	507nm	RCP Pump	143 ±14ps
*$CsPbBr_3$ NC SLs on glass, immersed in silicone oil (same NC batch as in dispersion)	509 nm	LCP Pump	357 ± 29 ps
*$CsPbBr_3$ NC SLs on glass, immersed in silicone oil (same NC batch as in dispersion)	509-510 nm	RCP Pump	619 ± 45 ps
$CsPbBr_3$ NCs in toluene	507 nm	LCP Pump	1.2 ± 0.1ps
$CsPbBr_3$ SLs on glass, immersed in silicon oil (same NC batch as in dispersion)	517 nm - 515 nm	LCP Pump	0.7 ± 0.1 ps

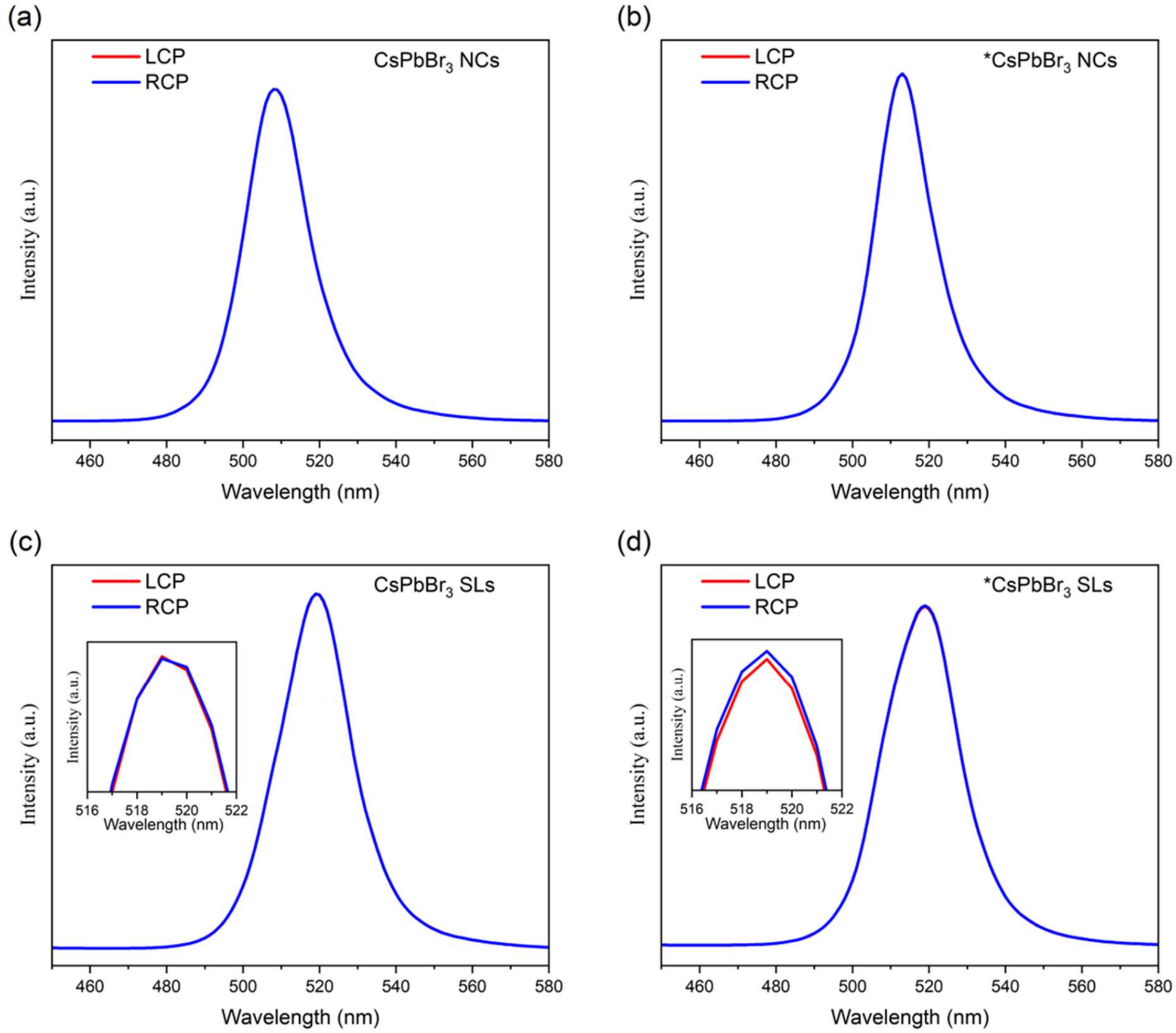

Figure S9. CPL intensity of the left and right circularly polarized light. **(a)** as-synthesized (achiral) $CsPbBr_3$ nanocrystals, **(b)** BCD-Br-treated $CsPbBr_3$ nanocrystals, **(c)** film of superlattices made from as-synthesized (achiral) $CsPbBr_3$ nanocrystals, and **(d)** film of superlattices made from BCD-Br-treated $CsPbBr_3$ nanocrystals. Insets in panels (c) and (d) show zoomed-in views of the spectra.

Note S2 Control CPL experiments

Additional CPL experiments to test the samples with a four-scan method. The measurements were performed in four sample orientations: front-side detection at 0° and 90° in-plane rotation, and back-side detection after flipping the sample at 0° and 90°. This protocol tests contributions from linear dichroism, linear birefringence, and sample-orientation artifacts.[10]

For CPL, we compared:

$g_{lum1} = [g\_{front}(0^o) + g\_{front}(90^o) + g\_{back}(0^o) + g\_{back}(90^o)]/4$, to characterize reciprocal component,

and

$g_{lum2} = [(g\_{front}(0^o) + g\_{front}(90^o)) - (g\_{back}(0^o) + g\_{back}(90^o)]/4$, to characterize non-reciprocal component.

For an achiral superlattice film sample, the g_{lum} values averaged across the PL spectrum (500-540 nm) are $g_{lum1} = (0.4 \pm 0.4)\times10^{-3}$, $g_{lum2} = -1.2 \pm 0.4\times10^{-3}$, establishing an effective detection limit. For an average chiral superlattice film sample, the corresponding values are $g_{lum1} = (1.3 \pm 0.3)\times10^{-3}$, $g_{lum2} = (-1.8 \pm 1)\times10^{-3}$. A measurably different reciprocal CPL component in the four-scan configuration confirms CPL in the BCD-Br-treated nanocrystal superlattices as compared to the achiral ones. A measurable non-reciprocal component indicates an LDLB contribution (in CD terminology). For CPL, this indicates a contribution from photoluminescence anisotropy and linear birefringence.

Table S2. Summarizing g_{lum} for superlattice films made from BCD-Br treated $CsPbBr_3$ nanocrystals from different individual batches and halide exchanged $CsPb(Cl/Br)_3$ or $CsPb(Br/I)_3$ nanocrystals.

**Superlattices (SLs)**	**g_{lum}**
*$CsPbBr_3$ SL 1 (Fig 4d)	0.003 ± 0.001
*$CsPbBr_3$ SL 2	0.004 ± 0.002
*$CsPbBr_3$ SL 3	0.004 ± 0.002
*$CsPbBr_3$ SL 4	0.006 ± 0.003
*$CsPbBr_3$ SL 5	0.007 ± 0.002
*$CsPb(Cl_{0.8}Br_{0.2})_3$	0.006 ± 0.002
*$CsPb(Br_{0.33}I_{0.67})_3$	0.003 ± 0.002

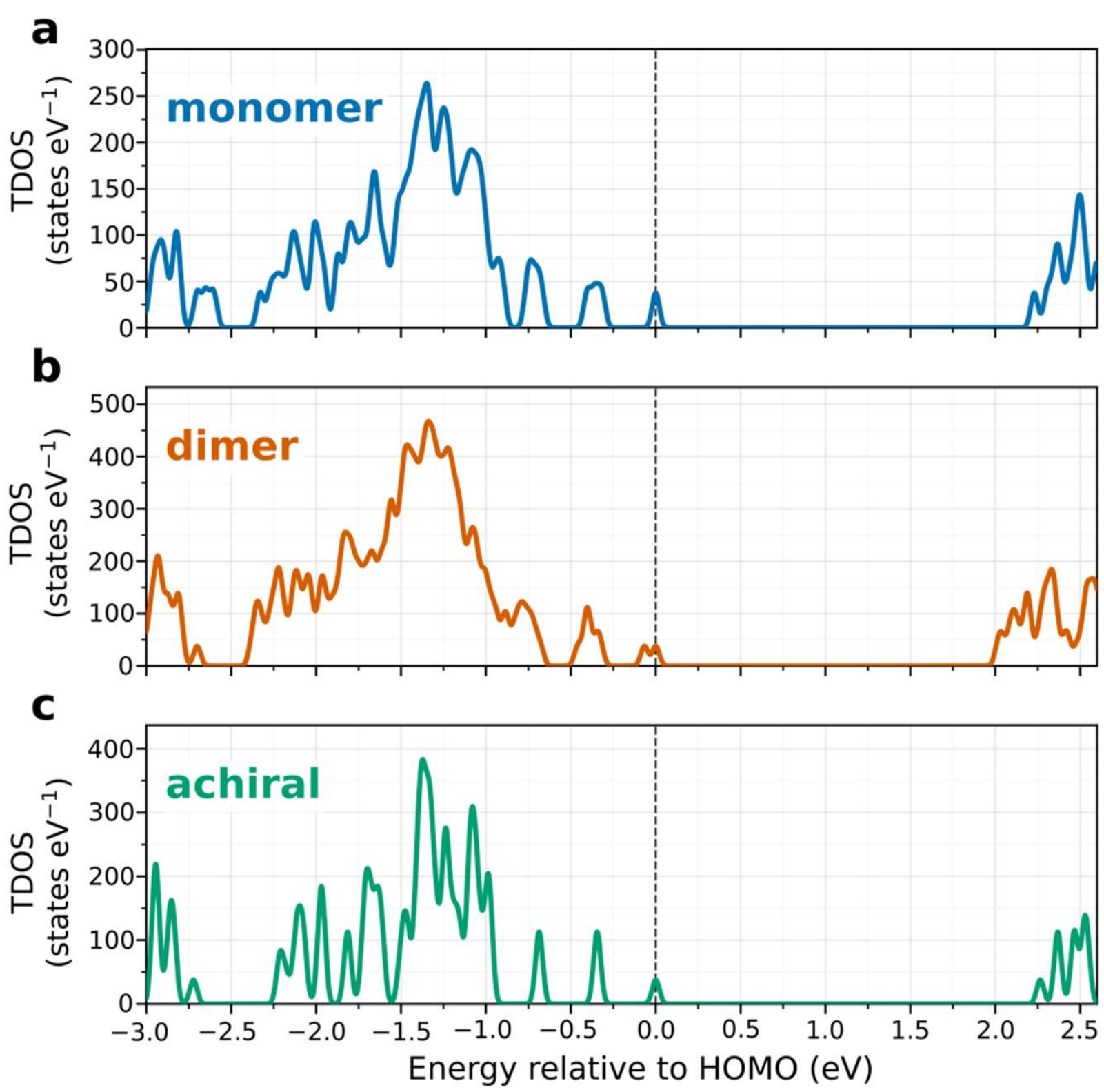

Figure S10. Comparison of the total density of states (TDOS) for (a) the chiral nanocrystal, (b) the chiral nanocrystal dimer, and (c) the achiral nanocrystal. The overall TDOS is similar for all three systems, with the achiral nanocrystal exhibiting sharper features owing to its higher structural symmetry. Only a modest reduction in the electronic gap is observed for the chiral nanocrystal dimer, consistent with the experimental observation of negligible spectral shifts upon superlattice formation.

References

1. Kresse, G.; Furthmüller, J., Efficient iterative schemes for ab initio total-energy calculations using a plane-wave basis set. *Phys. Rev. B* **1996,** *54*, 11169–11186.

2. Perdew, J. P.; Burke, K.; Ernzerhof, M., Generalized Gradient Approximation Made Simple. *Phys. Rev. Lett.* **1996,** *77*, 3865–3868.

3. Neese, F., Software Update: The ORCA Program System—Version 6.0. *WIREs Computational Molecular Science* **2025,** *15*, e70019.

4. Weigend, F.; Ahlrichs, R., Balanced basis sets of split valence, triple zeta valence and quadruple zeta valence quality for H to Rn: Design and assessment of accuracy. *Phys. Chem. Chem. Phys.* **2005,** *7*, 3297–3305.

5. Casida, M. E., Time-Dependent Density Functional Response Theory for Molecules. In *Recent Advances in Density Functional Methods*, WORLD SCIENTIFIC: 1995; Vol. Volume 1, pp 155–192.

6. Zaccaria, F.; Zhang, B.; Goldoni, L.; Imran, M.; Zito, J.; van Beek, B.; Lauciello, S.; De Trizio, L.; Manna, L.; Infante, I., The Reactivity of $CsPbBr_3$ Nanocrystals toward Acid/Base Ligands. *ACS Nano* **2022,** *16*, 1444–1455.

7. Charpentier, M., Asymmetric synthesis of myrtucommulone derivatives and synthesis of a biotin-linked myrtucommulone for affinity based target identification. *Saarland University* **2015**.

8. Toso, S.; Baranov, D.; Giannini, C.; Marras, S.; Manna, L., Wide-Angle X-ray Diffraction Evidence of Structural Coherence in CsPbBr3 Nanocrystal Superlattices. *ACS Materials Lett.* **2019,** *1*, 272–276.

9. Janzen, D. E.; Butler, M. S.; Reinheimer, E. W., Crystal structures of anhydrous and hydrated N-benzylcinchonidinium bromide. *Acta Crystallogr., Sect. E* **2022,** *78*, 594–598.

10. Albano, G.; Pescitelli, G.; Di Bari, L., Reciprocal and Non-reciprocal Chiroptical Features in Thin Films of Organic Dyes. *ChemNanoMat* **2022,** *8*, e202200219.
